# Knowledge, attitudes, and practices of pharmacy staff on cosmetic products in Lomé, Togo

**DOI:** 10.11604/pamj.2024.47.73.28986

**Published:** 2024-02-20

**Authors:** Julienne Noude Teclessou, Désiré Komla Amevor, Abla Séfako Akakpo, Abas Mouhari-Toure, Julie Zoua, Bayaki Saka, Koussake Kombate, Palokinam Pitche

**Affiliations:** 1 Dermatology Unit, Campus Teaching Hospital, University of Lomé, Lomé, Togo,; 2 Dermatology Unit, Sylvanus Olympio Teaching Hospital, University of Lomé, Lomé Togo,; 3 Dermatology Unit, Kara Teaching Hospital, University of Kara, Kara, Togo

**Keywords:** Knowledge, pharmacy staff, cosmetic

## To the Editors of the Pan African Medical Journal

Cosmetology is the art of making skin beautiful without transforming its internal nature. The use of cosmetics began a long time ago, mostly by women than men [1,2]. The use of cosmetics has increased these last decades due to modern methods of production and advertising. To ensure quality and preserve from side effects, patients are buying now cosmetics in the pharmacy. In Togo, the cosmetics in pharmacies are free-sale or after advice from pharmacists. This transversal, descriptive study conducted from 1^st^ June to 30^th^ November 2018 in private pharmacies of Lomé aimed to evaluate: the knowledge (definitions, types, uses, legislation, and regulation); the training on cosmetology; attitudes and practices of pharmacy workers concerning cosmetics users (advices, adverse effects report, guidance). The study included pharmacy sellers except cashiers. Each item was judged as bad if the rate of correct answers was inferior to 50%, insufficient (between 50-65%), average (65-85%), or good when correct answers were more than 85%. The Chi-carré test was used to compare variables.

Thirty of 44 identified pharmacies agreed to participate, summing up 148 participants. The average work experience of participants was 5.11 years (3 months to 20 years). Only 106 (71.6%) had basic medical training. The other basic training were management 8 (12.7%), office secretary 7 (11.1%), director assistant 7 (11.1%), sociology 5 (7.9%), none 7 (7.1%). Fifteen (10.13%) participants received training on cosmetology and 144 (77.0%) declared receiving a regular visit from a representative in pharmaceutical products who informed them about cosmetic products. The majority (92.7%) of participants confirmed receiving 1 to 10 cosmetic customers per week. The customers´ age ranged from 20-50 years and 96% of them were women. Twenty three percent of participants declared the regulating law of cosmetics sale in pharmacies, and 61.5% believed that cosmetics sold in pharmacies have fewer adverse effects on users. The definition of cosmetics as substances/mixtures designed to be applied on the skin was known by 130 (87.2%). The skin was the site of application of cosmetics according to 94.6% and its roles reported by participants were: to make beautiful (87.2%), to clean (85.1%), or to lighten (78.4%). The reasons for buying cosmetics by clients were respectively: remove stains (92.6%) and lighten the skin (89.9%). When clients wanted to buy cosmetics in pharmacies, 66.2% of participants advised them of their choice and 29.7% referred them to the dermatologists for a prescription. When the client demand was non-specific, 40.5% proposed a moisturizing perfumed cosmetic and 4.9% an enlightening one. The participants who had trained in cosmetology advised more moisturizing perfumed than non-perfumed cosmetics (p=0.006).

When a customer needed brightening cosmetics, only 31.7% of the surveys explained their adverse effects. Out of 109 respondents who received clients for adverse effects, 42.2% referred them to a dermatological consultation and 35.8% advised another lightening cosmetic. Globally, participants' knowledge of cosmetics was average like as their attitudes and practices about client´s needs. Also, the management of cosmetic adverse effects was low (Table 1). The main limitation of our study is that the results were not representative of all pharmacies in Lomé. The galenic forms of cosmetics were known by 95.3% of respondents. Ghiasi *et al*. in a similar study in Teheran found that 69.9% of participants had a good knowledge of the different cosmetics [3]. The roles of beautification (87.2%) and cleansing (85.1%) cited by the participants are the same as mentioned by the population who are using depigmenting cosmetics [2,4]. 91 (61.5%) participants believe that cosmetics sold in pharmacies have fewer adverse effects on users. Our study wasn´t interested in the sources of supply and quality of cosmetics; but wherever it is sold, the cosmetic´s adverse effects are the same [4,5].

**Table 1:** appreciation of knowledge, attitudes, and practices of pharmacy staff on cosmetic products in Lomé, Togo

	Good answer		Bad answer		Appreciation
	N	%	n	%	
**Knowledge**					
Definition of cosmetics	100	67.6	48	32.4	Average
Galenic forms	114	77.0	34	23.0	Average
Sites of application	98	66.2	50	33.8	Average
Role	119	80.4	29	19.6	Average
Laws on cosmetics	50	33.8	98	66.2	Bad
**Attitudes & practices**					
Advise in case of non-specific demand	111	75.0	37	25.0	Average
Faced with a lightening cosmetics demand	105	70.9	43	29.1	Average
Faced with lightening cosmetics side effects (n=109)	46	42.2	63	57.8	Poor

## Conclusion

Related to our results, we concluded that the training of pharmacy salesmen on cosmetics will be helpful for users to better manage them.
